# The Association of Psychological Variants with Back Pain, Muscle Endurance, and Functional Limitations in an Individual with Chronic Back Pain

**DOI:** 10.3390/jpm13121671

**Published:** 2023-11-29

**Authors:** Raee Saeed Alqhtani, Hashim Ahmed, Adel Alshahrani, Abdullah Mohammed Alyami, Abdur Raheem Khan, Ashfaque Khan

**Affiliations:** 1 Physiotherapy Program, Department of Medical Rehabilitation Sciences, College of Applied Medical Sciences, Najran University, Najran 55461, Saudi Arabia; rsalhyani@nu.edu.sa (R.S.A.); amsalshahrani@nu.edu.sa (A.A.); amalkhuriem@nu.edu.sa (A.M.A.); 2 Department of Physiotherapy, Integral University, Lucknow 226026, India; diriiahsr@iul.ac.in

**Keywords:** chronic back pain, psychological distress, muscle endurance, functional impairments

## Abstract

Chronic low back pain (CLBP) substantially impacts quality of life through a multifarious interplay of physical and psychological elements. A comprehensive understanding of this relationship is imperative for developing effective treatment strategies. This study recruited 64 participants (35 males and 29 females) experiencing chronic low back pain to explore the associations between psychological factors, muscle endurance, and functional impairments. The study was conducted over six months in an outpatient department and a rehabilitation unit. The study utilized established outcome measures, such as the Biering-Sorensen Test and the Roland Morris Disability Questionnaire, and psychological variants as the core dependent variables, including the Beck Depression Inventory (BDI), STAI questionnaire, the FABQ-PA, and the Pain Catastrophizing Scale (PCS). The findings uncovered pronounced gender disparities, with females exhibiting elevated levels of depression (BDI: 27.68 ± 9.43, *p* < 0.001) and anxiety (STAI: 42.34 ± 8.94, *p* < 0.001) and diminished muscle endurance (130.47 ± 30.56 sec, *p* = 0.001). These revelations are congruent with the prevailing literature, emphasizing the need for gender-sensitive and personalized interventions. Bivariate correlations presented robust associations between psychological distress and decreased muscle endurance (r values ranging from −0.82 to −0.88, *p* < 0.001) alongside elevated functional impairments (r values from 0.89 to 0.94, *p* < 0.001) for both genders. Additionally, linear regression analyses illuminated the consequential impact of specific psychological variables such as the BDI, FABQ-PA, and PCS on muscle endurance and functional impairments (all *p* < 0.001). This study reveals gender-specific variations in chronic back pain, highlighting the influence of psychological factors on pain perception. It underscores the necessity for gender-sensitive treatment strategies. Future research is needed to explore these differences further and assess treatment efficacy to improve care and quality of life for chronic low back pain sufferers through personalized treatment plans.

## 1. Introduction

Chronic low back pain (CLBP) is a prevalent condition that affects a significant number of individuals across the globe, causing a substantial reduction in their quality of life. [1]. The manifestation of CLBP is influenced by a combination of physical and psychological factors. These factors encompass a range of emotions such as depression, anxiety, fear-avoidance, and pain catastrophizing. Their impact is crucial in terms of the initiation, continuation, and intensity of pain [2,3]. The primary objective of this research is to gain a deeper understanding of the correlation between psychological factors and the occurrences of chronic back pain, muscle endurance, and functional impairments. Through this study, we aim to examine the root causes of these conditions and identify potential interventions that may help alleviate the associated symptoms.

CLBP is a significant issue for public health. It is one of the primary causes of disability across the globe and often leads to medical consultations and absenteeism from work. The economic burden of CLBP is substantial, including direct medical expenses and indirect costs related to lost productivity and disability. Moreover, the impact of CLBP goes beyond the individual, affecting families, communities, and healthcare systems.

Chronic low back pain is linked to various functional limitations and a decrease in muscle endurance, which perpetuates a cycle of pain and disability [4]. A thorough understanding of these relationships is essential for devising targeted interventions and enhancing the well-being of individuals grappling with chronic back pain [5]. Moreover, examining psychological factors is paramount as their substantial contribution to the chronicity and severity of back pain has been well established [6].

The multifactorial nature of CLBP complicates its management. It involves treating physical symptoms and addressing psychological, social, and environmental factors. The biopsychosocial model of pain provides a framework for understanding these complex interactions and emphasizes a holistic approach to pain management.

Individuals suffering from chronic back pain have been shown to exhibit elevated levels of psychological distress compared with the general populace [7]. Depression, characterized by persistent sadness and hopelessness, closely intertwines with chronic back pain, influencing pain perception and leading to functional limitations [8]. Anxiety exacerbates pain perception and encourages behaviors that can further contribute to disability [9]. The interplay between psychological distress and chronic back pain underscores the importance of addressing both mental and physical health [10].

Emerging research suggests that lifestyle factors, such as diet, exercise, sleep, and stress management, can significantly contribute to the onset and maintenance of the musculoskeletal system [11]. Utilizing interventions that address these lifestyle factors in conjunction with traditional medical treatments could provide a more comprehensive approach to managing CLBP.

Evidence suggests that addressing both the psychological and physical aspects through interventions like cognitive behavioral therapy can alleviate distress and improve outcomes [12]. Such integrated approaches highlight the potential for more comprehensive management of chronic back pain [13]. Yet despite these advancements, limitations persist, necessitating the further exploration and refinement of treatment modalities [14].

In recent years, there has been growing interest in using technology-based interventions for CLBP, such as telerehabilitation and digital health apps. These interventions offer new possibilities for managing CLBP, particularly regarding accessibility and personalized care.

The objective of this study is to investigate the association between psychological factors and the physical symptoms of chronic low back pain. To achieve this goal, widely recognized assessments like the Beck Depression Inventory (BDI), the State-Trait Anxiety Inventory (STAI-Y), the Fear Avoidance Beliefs Questionnaire (FABQ), and the Pain Catastrophizing Scale (PCS) [13,14,15,16,17,18] are utilized to evaluate psychological factors. The ultimate objective is to provide valuable insights that can be applied to improve current knowledge and establish more comprehensive and effective treatments [15].

Another important aspect is the role of healthcare professionals in managing CLBP. Multidisciplinary teams, including physicians, physiotherapists, psychologists, and occupational therapists, are essential for comprehensive care. Training healthcare providers to recognize and address the psychological aspects of CLBP is crucial.

Given the profound impact of chronic low back pain on individuals and healthcare systems, exploring the multifaceted relationships between psychological factors, muscle endurance, and functional impairments is of paramount importance [16]. By examining these intricate relationships, this research seeks to address existing gaps in knowledge, offering solutions and shedding light on potential avenues for future research and intervention strategies [17]. Through this comprehensive examination, we aim to enhance understanding, propose optimal solutions, and ultimately alleviate the burden of chronic back pain on affected individuals [18].

## 2. Methods

### 2.1. Study Design

A cross-sectional observational design was employed in the current investigation in which sixty-four participants suffering from chronic low back pain were strategically recruited through a convenience sampling method, ensuring a diverse representation of demographics.

### 2.2. Ethical Considerations

Approval for this study was granted by the University’s Research Ethics Committee (IEC/IIMS/2022/70), confirming adherence to the Declaration of Helsinki and safeguarding the rights and well-being of the participants. Every participant was briefed on the study’s aims and procedures, following which written informed consent was secured [19].

### 2.3. Setting

This research was conducted in the outpatient department and rehabilitation unit of the Integral Hospital and Research Center over a six-month period between 1 March 2023, and 31 August 2023. Participants had stable vital signs consistently reflected in measurements taken at various intervals. Demographic information, including age, gender, marital status, education level, socioeconomic status, living conditions, and social status, was thoroughly documented before screening. A total of sixty-four participants suffering from chronic low back pain were meticulously recruited for this cross-sectional observational study. The sample size was determined using the formula $n=z^{2}p(1-p)/d^{2}$, where *p* = 50.0%, using Z (1.65) at a 90% confidence interval and an error of 10.3% to get a sample size of 64 [20]. Participants underwent a rigorous screening process, and only those who met the predetermined inclusion criteria were enlisted for the study. The inclusion criteria were clearly defined: individuals aged between 18 and 65 years diagnosed with chronic low back pain persisting for at least six months. Those who did not meet the inclusion criteria or corresponded with any of the exclusion criteria were systematically excluded from the study [21]. The exclusion criteria comprised individuals with significant co-morbidities, cognitive impairments, or those expressing unwillingness to participate in the study. Every measure was taken to ensure the ethical and inclusive selection of participants, fostering a diverse and representative sample for the study.

### 2.4. Variables

This study emphasized psychological variants as the core dependent variables, focusing specifically on depression, anxiety, fear avoidance, and pain catastrophizing. Each of these psychological elements was meticulously measured to gain a profound understanding of their impact and interconnections.

**Depression:** The evaluation of depression levels in this study relied on the Beck Depression Inventory (BDI), a widely used tool in the field of psychology. The BDI entailed a structured questionnaire consisting of 21 questions that were designed to carefully measure the severity of depression among participants. The participants were asked to select the statement that accurately reflected their emotions over the past two weeks, including the day of the assessment. Based on the *Diagnostic and Statistical Manual of Mental Disorders*, Fourth Edition, the BDI scores were then categorized as minimal, mild, moderate, or severe. The BDI exhibited high internal consistency, with a mean alpha coefficient of 0.86 among psychiatric patients and 0.81 among non-psychiatric individuals. This indicates that the BDI is a reliable and valid assessment tool for detecting depression in both clinical and non-clinical settings. The participants were thus able to provide accurate and consistent responses that allowed for a thorough evaluation of their depression levels [22,23].

**Anxiety:** We used the STAI questionnaire to evaluate the participants’ anxiety levels. This inventory is designed to be self-administered and consists of 20 questions that target specific aspects of anxiety. The theory behind this tool suggests that anxiety is a temporary emotional state characterized by feelings of apprehension, tension, and increased autonomic nervous system activity that are consciously perceived. Before completing the questionnaire, participants were given clear instructions on its purpose and method. They were then asked to rate how much each statement reflected their current anxiety levels. The resulting scores could range from 20 to 80, with lower scores indicating minimal anxiety and higher scores indicating heightened anxiety. Clinically, a score ranging from 39 to 40 is considered the optimal cut-off for significant state anxiety symptoms. In contrast, a score between 44 and 50 denotes a chronically ill patient suffering from an anxiety disorder [24].

**Fear Avoidance:** In order to assess individuals’ fear-avoidance beliefs related to physical activity, the FABQ-PA questionnaire was utilized. A modified questionnaire was tailored to the participants’ experiences and perceptions, focusing on standing, walking, stepping, and the stand-to-sit transition rather than the original lower-back section activities of bending, lifting, walking, and driving. The FABQ-PA measures fear levels associated with physical activities and assesses beliefs regarding the relationship between pain and such activities through four items. Each item was rated on a detailed 7-point scale ranging from “completely disagree” (0) to “strongly agree” (6), with “unsure” serving as the neutral anchor term. The highest cumulative score possible was 24. A therapist guided participants through the questionnaire and rated their fear, avoidance attitudes, and beliefs regarding physical activities. Higher scores indicated higher fear-avoidance beliefs, with a score above 15 indicating elevated levels. Scores on the FABQ-PA ranged from 0 to 24 [25].

**Pain Catastrophizing:** Pain Catastrophizing was measured using the Pain Catastrophizing Scale (PCS), a widely recognized tool comprising 13 items. This scale assesses three distinct dimensions of catastrophizing: rumination, helplessness, and magnification. Rumination is evaluated through four specific questions (items 8–11), helplessness through six questions (items 1–5 and 12), and magnification through three questions (items 6, 7, and 13). Each item on the scale is rated on a 5-point Likert scale ranging from 0 (not at all) to 4 (all the time), allowing for a nuanced understanding of the patient’s catastrophizing tendencies. The total score is calculated by summing the responses to all 13 items, with the potential score range being from 0 to 52. Higher scores on the PCS indicate more significant levels of pain catastrophizing, providing valuable insight into the patient’s psychological coping mechanisms with their pain experience. This tool is essential in understanding the psychological aspects of pain management and helps in tailoring appropriate therapeutic interventions [26].

### 2.5. Muscle Endurance

Muscle endurance in this study was assessed using the Biering-Sorensen test, a well-established method for evaluating the endurance of trunk extensor muscles. During the test, participants lie prone, maintaining their upper body suspended over the edge of a plinth while the lower body remains supported. The objective is to hold this position for as long as possible, with the duration in seconds being recorded. This duration reflects the isometric endurance capacity of the individual’s trunk extensor muscles. The Biering-Sorensen test is a widely recognized and validated tool which is particularly effective for assessing muscle endurance in individuals experiencing low back pain. Its application in this study provides a reliable measure of muscle endurance, offering valuable insights into the physical capabilities and limitations of participants suffering from low back pain [27].

### 2.6. Functional Impairment

Functional impairment was evaluated in the participants using the Roland Morris Disability Questionnaire (RMDQ), a comprehensive 24-item self-report questionnaire specifically designed to measure the degree of physical disability attributable to low back pain. This questionnaire encompasses a range of daily activities and tasks, with each item reflecting a different aspect of functional ability affected by back pain. Participants responded to these items, indicating their current ability or difficulty performing these activities. The total score, derived from the sum of all items, ranges so that higher scores indicate greater functional impairment. The RMDQ is widely recognized for its reliability and validity in assessing the functional status of individuals suffering from low back pain, making it a valuable tool for this study. It helps in quantifying the extent of disability, thereby offering a clear picture of how low back pain affects day-to-day functioning [28].

## 3. Data Analysis

The collected data were meticulously analyzed using SPSS version 22.0 (IBM Corporation, Armonk, NY, USA). For comparisons between psychological variables, measures of function, and physical performance between different groups, the independent *t*-test was employed when the assumption of normal distribution within each group and approximately equal variances of the two groups was met. Conversely, in cases in which these assumptions were not satisfied, the Mann–Whitney U test was utilized. The PPMCC was utilized to assess the association among psychological variables, function measures, and physical performance for normally distributed continuous variables. Further, a series of linear regression analyses were conducted to evaluate the relationships and interaction effects among psychological variables on measures of function and physical performance. Statistically significant differences were identified when *p* was less than or equal to 0.05.

## 4. Results

The total calculated sample size was 64. In total, 35 males and 29 females suffering from chronic low back pain were successfully included in the study. We only included participants who fulfilled the inclusion criteria. Seven participants declined due to unavailability. Therefore, we conducted a statistical analysis on the remaining 57 study participants. The participants had a response rate of 78.05%, and their data are expressed as means and standard deviations (SDs) where applicable. The mean ages of the females and males in this study were 34.5 ± 7.2 years and 32.8 ± 6.9 years, respectively.

Table 1 presents a comparative analysis between male and female participants regarding psychological variables, muscle endurance, and functional impairments. The psychological variables studied include depression (BDI), anxiety (STAI), fear avoidance (FABQ-PA), and pain catastrophizing (PCS). The table provides mean scores and standard deviations for each group, along with the T values, degrees of freedom (Dfs), and *p*-values from independent *t*-tests, enabling a comparison between gender groups. The results reveal statistically significant differences (*p* < 0.05) between males and females for all listed variables, with females demonstrating higher scores for psychological variables and functional impairments and lower scores for muscle endurance. The significant *p*-values are indicative of robust differences in the experience and manifestation of chronic back pain between the genders.

Table 2 illustrates the significant bivariate correlations between selected psychological variables (depression, anxiety, fear avoidance, and pain catastrophizing) and outcomes of muscle endurance and functional impairments. The negative correlations with muscle endurance and positive correlations with functional impairments suggest that higher levels of these psychological factors are associated with decreased muscle endurance and increased functional impairments. All relationships are significant at the 0.001 level, indicating strong and statistically significant correlations between the examined psychological variables and physical outcomes.

Table 3 shows the gender-based bivariate correlations between the psychological variables, namely depression (BDI), anxiety (STAI), fear avoidance (FABQ-PA), and pain catastrophizing (PCS), and the measures of muscle endurance and functional impairments. For both males and females, the psychological variables exhibit negative correlations with muscle endurance and positive correlations with functional impairments, indicating that higher psychological distress is associated with lower muscle endurance and higher functional impairments within each gender. Notably, all correlations were found to be statistically significant at the 0.001 level for both genders, suggesting robust relationships between the psychological factors and the measures of physical outcomes across both male and female populations.

Table 4 displays the results of a linear regression analysis exploring the impact of the psychological variables (BDI, STAI, FABQ-PA, and PCS) on muscle endurance and functional impairments. The Y-values represent the regression coefficients, indicating the change in the dependent variable associated with a one-unit change in the independent variable, holding other variables constant. The *p*-values signify the statistical significance of the relationships.

For muscle endurance, the table reveals statistically significant negative relationships with the BDI, FABQ-PA, and PCS scores (all *p* < 0.001), suggesting these variables contribute to decreased muscle endurance. However, the STAI score showed no significant impact (*p* = 0.615).

In terms of functional impairments, significant negative relationships were observed with the BDI and PCS (both *p* < 0.001), while the STAI and FABQ-PA did not significantly affect the functional impairments (*p* = 0.56 and *p* = 0.63, respectively).

## 5. Discussion

The present study aimed to examine the association between psychological factors and their impact on muscle endurance and functional deficits in individuals suffering from chronic back pain, providing insights into various important elements. The findings of this study are consistent with the existing body of literature, highlighting the significant influence of psychological factors on the development and physical consequences of chronic back pain.

One notable observation derived from our study is the significant disparities observed between male and female subjects in relation to psychological distress and the physical manifestations thereof in the context of chronic back pain. Female individuals exhibited heightened degrees of depression, anxiety, fear avoidance, and pain catastrophizing. As a result, this particular group had decreased muscle endurance and increased functional deficits. The observed gender-specific inequalities in psychological distress and its repercussions in chronic pain scenarios align with the findings of a study conducted by Sullivan et al. [29].

Further delving into the core findings, the study revealed robust and significant correlations between psychological factors and both muscle endurance and functional impairments across genders. This observation supports the assertions made by Butt and Trevor, which found a direct correlation between increased psychological distress and diminished muscle endurance and escalated functional impairments [30]. In particular, the study found substantial evidence supporting the significant impact of depression and pain catastrophizing on physical outcomes, consistent with the findings of William et al., emphasizing their role in the context of chronic back pain [31].

Many studies indicated the differences between men and women regarding symptomatology and their reactions or handling. Most of the studies reported that females have higher pain acceptance levels and are less afraid of pain [32]. They reported higher activity levels and activity engagement despite having the same level of pain, the same severity of symptoms, the same discomfort, and the same somatic health as men. However, men scored having higher physical function but also struggling with psychological issues, such as mood disturbances. Thus, indicating if outcomes were potentially sex-dependent, treatment may be more effective by differentially targeting the processes supporting the different needs, expectations, and coping mechanisms of each gender. Clinical and rehabilitation-relevant measures are those sensitive to genders’ potentially differential rehabilitation needs. These differences may also support decision-making to assess and allocate patients to different rehabilitation programs potentially designed for the needs of the different genders.

However, the results regarding anxiety and fear avoidance present a deviation from some earlier studies. Despite significant correlations with muscle endurance and functional impairments in the bivariate analysis, these variables did not have significance in the linear regression model. This difference may suggest a more complex and multifaceted interaction between these variables than previously considered, as noted by Dymond S et al. [33].

Assessing the methodological approach employed, the combination of established inventories and statistical analysis carried out through SPSS was crucial in unravelling the intricate relationships under investigation [13]. The adoption of validated measures such as the Beck Depression Inventory (BDI), State-Trait Anxiety Inventory (STAI-Y), Fear-Avoidance Beliefs Questionnaire (FABQ), and Pain Catastrophizing Scale (PCS) enhanced the reliability and depth of our insights.

Nevertheless, certain limitations need to be addressed. The sample size, albeit sufficient for this study, limits the generalizability of the findings to a wider demographic. Future research with a more diverse and larger participant pool is essential to further validate and expand upon these findings. Additionally, the cross-sectional design of this study poses limitations in drawing causal relationships between the studied variables, emphasizing the need for longitudinal studies for a more thorough understanding.

This study provides valuable insights into the significant role of psychological factors in chronic back pain and their substantial associations with muscle endurance and functional impairments. The outcomes underline the importance of integrating psychological interventions in chronic back pain management and underscore potential directions for further research and the development of comprehensive treatment strategies. Despite the limitations, the study contributes significantly to the growing literature, highlighting the need for an integrated approach to address the multifaceted nature of chronic back pain.

The gender-specific disparities observed in our study have significant implications for clinical practice. Considering these differences, it would be beneficial to tailor interventions such as emotional training, psychological support, and psychological consultancy to cater specifically to the unique needs of each gender. For instance, females exhibiting higher levels of depression and anxiety might benefit more from therapeutic approaches focusing on emotional regulation and stress management. Conversely, men who show higher physical function but struggle with mood disturbances might require more targeted psychological support to address these issues. Implementing evidence-based practices that acknowledge these gender-specific needs could enhance the effectiveness of chronic back pain management, leading to better patient outcomes.

## 6. Conclusions

This study sheds light on gender-specific differences in chronic back pain and suggests the potential role of psychological factors in pain perception. These insights contribute to understanding this complex condition and underline the need for tailored treatment strategies sensitive to gender differences. While this research offers essential directions for personalized care, future investigations are warranted to explore the causes of these differences and evaluate the long-term efficacy of various therapeutic approaches. Hopefully, this study will aid in enhancing patient care and quality of life for those suffering from chronic low back pain, emphasizing the importance of individualized treatment plans.

## Figures and Tables

**Table 1:** Comparative analysis of psychological variables, muscle endurance, and functional impairments between males and females.

Variable	Male	Female	T Value	df	*p*-Value
Depression (BDI)	19.23 ± 8.76	27.68 ± 9.43	4.63	55	<0.001
Anxiety (STAI)	35.45 ± 7.11	42.34 ± 8.94	3.76	55	0.001
Fear Avoidance (FABQ-PA)	10.67 ± 6.21	18.43 ± 7.91	5.11	55	<0.001
Pain Catastrophizing (PCS)	20.45 ± 9.12	30.58 ± 10.47	4.82	55	<0.001
Muscle Endurance	150.32 ± 35.67 s	130.47 ± 30.56 s	2.78	55	0.001
Functional Impairments	7.45 ± 3.12	12.34 ± 4.76	5.43	55	<0.001

**Table 2:** Bivariate correlation between psychological variables and measures of muscle endurance and functional impairments.

Variable	Muscle Endurance		Functional Impairments	
	r Value	*p*-Value	r Value	*p*-Value
Depression (BDI)	−0.87 *	<0.001 *	0.92 *	<0.001 *
Anxiety (STAI)	−0.82 *	<0.001 *	0.89 *	<0.001 *
Fear Avoidance (FABQ-PA)	−0.85 *	<0.001 *	0.91 *	<0.001 *
Pain Catastrophizing (PCS)	−0.88 *	<0.001 *	0.94 *	<0.001 *

*: significant correlation.

**Table 3:** Gender-based bivariate correlation between psychological variables and measures of muscle endurance and functional impairments.

Variable	Muscle Endurance		Functional Impairments	
	r Value	*p*-Value	r Value	*p*-Value
Depression (BDI)				
Male	−0.87 *	<0.001 *	0.92 *	<0.001 *
Female	−0.99 *	<0.001 *	0.95 *	<0.001 *
Anxiety (STAI)				
Male	−0.82 *	<0.001 *	0.89 *	<0.001 *
Female	−0.99 *	<0.001 *	0.93 *	<0.001 *
Fear Avoidance (FABQ-PA)				
Male	−0.85 *	<0.001 *	0.91 *	<0.001 *
Female	−0.98 *	<0.001 *	0.94 *	<0.001 *
Pain Catastrophizing (PCS)				
Male	−0.88 *	<0.001 *	0.94 *	<0.001 *
Female	−0.99 *	<0.001 *	0.97 *	<0.001 *

*: significant correlation.

**Table 4:** Linear regression analysis of psychological variables impacting muscle endurance and functional impairments.

Variable		Linear Regression	
Independent	Dependent	Y-Value	*p*-Value
Muscle Endurance	BDI	−0.329 **	<0.001 **
	STAI	−0.043 ns	0.615 ns
	FABQ-PA	−0.591 **	<0.001 **
	PCS	−0.410 **	<0.001 **
Functional Impairments	BDI	−0.319 **	<0.001 **
	STAI	−0.052 ns	0.56 ns
	FABQ-PA	−0.049 ns	0.63 ns
	PCS	−0.375 **	<0.001 **

**: significant correlation; ns: non-significant correlation

## Data Availability

The datasets analyzed in the current study are available from the corresponding author upon reasonable request.
